# The Critical Impact of Sub‐ and Supraoptimal Temperatures on Male Fertility Potential of an Invasive Fruit Fly

**DOI:** 10.1002/ece3.71515

**Published:** 2025-06-04

**Authors:** Hervé Colinet, Coline Lehnhoff, Bréa Raynaud‐Berton

**Affiliations:** ^1^ Université de Rennes, CNRS, ECOBIO (Ecosystèmes, Biodiversité, Évolution) Rennes France

**Keywords:** drosophila, males, mating, thermal performance curves, winter acclimation

## Abstract

In insects, thermal fertility limits have a narrower tolerance range than survival. Therefore, deciphering these critical limits is crucial for understanding population dynamics during or after harsh winter or summer periods. Here investigated the impact of temperature on male fertility in the invasive pest, *Drosophila suzukii*. We assessed both developmental and postemergence temperatures, using a range of 11 temperatures from 10°C to 30°C and varying chronic exposure durations. The results revealed asymmetrical thermal performance curves for male fertility, with thermal fertility limits (TFLmin and TFLmax) between 9.8°C and 29°C for developmental temperatures and between 10.05°C and 34.8°C for adult temperatures. Males developed at sub‐ or supraoptimal temperatures were sterile at emergence, and no recovery occurred if the temperature was maintained. Males developed and maintained at supra‐optimal temperatures were more sensitive than those exposed to supra‐optimal conditions only during the adult stage, highlighting the cumulative effects of thermal stress across life stages. Supra‐optimal temperatures only at the adult stage induced some sterility in initially fertile males after a few days, whereas males exposed to suboptimal temperatures never reached fertility, remaining sterile throughout the experiment. Males that underwent winter‐like cold‐acclimation were in reproductive quiescence and progressively regained fertility when transferred to 20°C. Morphometric analyses of reproductive organs showed that cold‐acclimated winter males had anatomical traits similar to the controls after 12 days of recovery. Despite this, mating tests indicated that cold‐acclimated males were less attractive to females, suggesting a cost on reproductive potential. This study defines the thermal limits for male sterility under chronic exposure and highlights a partial recovery of fertility in cold‐acclimated winter males. It thus provides key insights into the population dynamics of *D. suzukii* after winter.

## Graphical abstract

This study investigates the thermal limits of male fertility in Drosophila suzukii under varying temperature conditions. We assess the fertility of males developed at different temperatures, including the effects of cold acclimation and subsequent temperature shifts, as well as reproductive success and organ development. Our findings provide key insights into the impact of temperature on reproductive success and the population dynamics of this invasive pest.

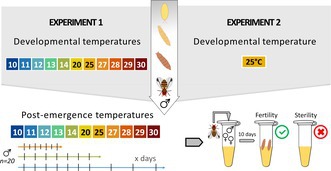

## 1 Introduction

Reproduction is as crucial as survival for any living organism, ensuring the continuity of species, genetic heredity, and population sustainability. While survival ensures that organisms can live through the challenges of their environment, it is fertility—defined by reproductive success and the ability to produce viable offspring—that directly influences population dynamics and species persistence (Genovart et al. 2018; Southwood and Comins 1976). Unlike fecundity, which measures the potential reproductive capacity through the number of offspring produced, fertility provides a direct link to reproductive success and long‐term population viability (Leridon 2007).

Ectothermic species like insects are highly vulnerable to temperature fluctuations due to their small size and reliance on external heat sources for thermoregulation, with cooler refuges playing a key role in preventing overheating during summer (Buckley and Huey 2016; Clusella‐Trullas et al. 2011). Changes in critical thermal limits for survival (CTmin, CTmax) significantly affect their geographical distribution, reproduction, and overall fitness (Moore et al. 2023). Even in relatively stable environments, temperature fluctuations can pose significant challenges (Colinet et al. 2015). Although thermal tolerance, often assessed through critical thermal limits, has traditionally been used to predict species persistence, recent studies suggest that reproductive thermal limits may provide a more accurate predictor of vulnerability. These limits could offer a finer measure of species' thermal susceptibility than survival (Grandela et al. 2024; Ørsted et al. 2024; Parratt et al. 2021; Van Heerwaarden and Sgrò 2021; Walsh et al. 2019).

In addition to the thermal stress caused by climate change, insects must also face the annual challenge of seasonality including overwintering. To survive winter, insects display a range of responses from long‐term adaptation to more rapid phenotypic adjustments (Colinet and Hoffmann 2012; Lee 2010; Toxopeus and Sinclair 2018). Insects exhibit various forms of dormancy as part of their seasonal responses, ranging from deep dormancy (e.g., diapause) to lighter states of inactivity (Denlinger 2002; Kubrak et al. 2014, 2016; Toxopeus and Sinclair 2018). In some species, adults undergo a reversible reproductive arrest (i.e., reproductive dormancy) under certain thermal thresholds, allowing energy from reproduction to be reallocated to other functions, such as survival (e.g., Colinet et al. 2012; Lirakis et al. 2018; Mensch et al. 2016). Therefore, studying reproductive thermal limits is crucial for predicting extinction risk under climate change, but also for understanding population dynamics during or after harsh winter or summer periods when reproductive activity can be halted (Kreiman et al. 2023; Panel et al. 2020).

This study focuses on the spotted wing *Drosophila* (*Drosophila suzukii*), an invasive species originating from Southeast Asia and that is now distributed worldwide, causing significant agricultural damage (Tait et al. 2021). The phenotypic plasticity of *D. suzukii* allows flies to tolerate various abiotic stressors, including temperature extremes (Little et al. 2020; Winkler et al. 2020), making this system an ideal models to study thermal effects on fertility. Although the female's reproductive traits of *D. suzukii* have been well‐studied due to the direct impact of oviposition on fruit crops (Tait et al. 2021), the males' reproductive traits remain underexplored (Green et al. 2019). Evidence from related species, such as *Drosophila melanogaster* , suggested that temperature can significantly affect males and their spermatogenesis (Chakir et al. 2002; David et al. 2004; Kubrak et al. 2016; Meena, Maggu, et al. 2024). Overall, across taxa, spermatogenesis seems more susceptible to temperature stress than oogenesis (Kreiman et al. 2023; Iossa 2019; Meena, Maggu, et al. 2024; Sales et al. 2018; Wang and Gunderson 2022; Zwoinska et al. 2020) making male fertility a key component of population fitness to assess in the context of both overwintering and climate change. However, in *D. suzukii*, the specific thermal thresholds for male sterility at both high and low temperatures, as well as the ability of males to recover fertility after cold‐induced reproductive arrest in the dormant phenotypes, remain to be characterized.

Seasonal variations in fertility are common in *Drosophila* species, cold‐hardy flies tend to have high fertility in spring and low fertility in autumn, reflecting the adaptation to fluctuating environments (Grechanyĭ et al. 1997). In *D. suzukii*, females are considered the primary overwintering sex, capable of surviving unfavorable seasonal conditions by reversibly halting oogenesis and resuming it in spring (Sario et al. 2023; Stephens et al. 2015; Winkler et al. 2020). This phenomenon is temperature‐dependent and not necessarily regulated by photoperiod, indicating a reproductive quiescence rather than true diapause (Colinet and Kustre 2025; Toxopeus et al. 2016). In other *Drosophila* species, gametogenesis has also been shown to be reversibly halted in response to low temperature (e.g., Chakir et al. 2002; David et al. 2004; Lirakis et al. 2018; Kubrak et al. 2014; Mensch et al. 2016; Salminen and Hoikkala 2013; Vollmer et al. 2004). While such cold‐induced reproductive arrest has been well‐documented in females, knowledge regarding males remains scarcer (Kubrak et al. 2016). In particular, in *D. suzukii* there is limited understanding of how males maintain or recover fertility after winter conditions. Only 50% of males *D. suzukii* collected in winter and spring had sperm in their testes, suggesting a seasonal decrease or arrest in spermatogenesis (Grassi et al. 2018). Generally speaking, little is known about the overwintering of male *D. suzukii*, including whether they enter reproductive dormancy as well as their capacity to recover from gametogenesis arrest.

Some *Drosophila* studies have explored the fertility recovery after thermal stress. However, this process has been almost exclusively analyzed in the context of heat stress, rather than recovery from suboptimal temperatures and/or recovery after cold‐induced reproductive dormancy (e.g., Canal Domenech and Fricke 2022; Green et al. 2019; Jørgensen et al. 2006; Kubrak et al. 2014, 2016; Nguyen et al. 2013; Sales et al. 2021). Understanding male fertility in response to thermal conditions could provide insights into population dynamics and pest management strategies throughout the year. Most studies on insect fertility in response to temperature have focused on exposures either at the adult stage or during development. However, recent studies have begun to examine conditions encompassing both developmental and adult stages (e.g., Canal Domenech and Fricke 2022; Meena, De Nardo, et al. 2024). Still, to our knowledge, few studies have systematically investigated both temperature extremes (heat and cold) across these stages (but see Araripe et al. 2004; Chakir et al. 2002). In particular, there is considerable interest in heat‐induced sterility due to climate change concerns (e.g., Grandela et al. 2024; Parratt et al. 2021; Van Heerwaarden and Sgrò 2021; Walsh et al. 2019), while the effects of low temperatures have been disproportionately understudied. Moreover, most studies generally use a limited number of temperature, making it hard to identify thermal optima and limits (Kingsolver et al. 2014). As recently emphasized by Wang and Gunderson (2022), data on male reproductive potential are rarely discussed within the context of performance curves, despite the fact that this approach is essential for a comprehensive understanding of reproductive traits in animals.

In this study, we used a wide range of temperatures and durations to explore how male fertility varies with temperature in different life stages of *D. suzukii*. We modeled the thermal performance curve (TPC) for male fertility according to a range of developmental and adult temperatures to identify cold‐ and heat‐induced fertility thresholds. We hypothesized that if thermal fertility limits (TFLs) have a narrower tolerance range than survival (see Parratt et al. 2021; Van Heerwaarden and Sgrò 2021; Walsh et al. 2019), then the lower and upper thermal fertility limits (i.e., TFLmin and TFLmax) should be respectively higher and lower than the viability limits. We also assumed that cold‐induced male sterility will be within the range of values reported in other *Drosophila* species, typically 10°C–14°C (e.g., Araripe et al. 2004; Chakir et al. 2002; Vollmer et al. 2004; Meena, Maggu, et al. 2024). In insects, cumulative heat exposure up to the pupal stage is particularly detrimental, due to the high sensitivity of the last steps of spermatogenesis occurring during the pupal stage (Canal Domenech and Fricke 2023; Meena, Maggu, et al. 2024; Sales et al. 2021). Thus, we hypothesized that exposure to thermal stress during whole development may lead to lasting detrimental effects on fertility, and that exposure to sub‐ or supraoptimal temperatures during both developmental and adult stages (Experiment 1) will have a cumulative negative impact on reproductive performance. In contrast, exposure to sub‐ or supraoptimal temperatures exclusively during the adult stage (Experiment 2) would have less of an impact on fertility, indirectly suggesting that adult‐stage flies may have a greater capacity for thermal recovery. In a second experiment, we thus investigated male fertility by exposing adults to a range of chronic low and high temperatures for various durations. When males emerge, spermatozoids are already present but not yet mobile and accumulated in the seminal vesicle (Perrin‐Waldemer 1966). We hypothesized that chronically exposing males to temperatures outside the optimal fertility range during sexual maturation (i.e., after emergence) may progressively reduce sperm production and viability, thereby decreasing fertility, even if eclosed males were fertile prior to exposure. Finally, to assess the reproductive capacity of overwintering *D. suzukii* males, we reared and acclimated males at low temperature (hereafter the winter‐acclimated phenotype: WA) and evaluated their fertility and its recovery, mating competitiveness, and size and color of reproductive organs at various time points. In insects, males exposed to sub‐ or supraoptimal temperatures are often at a disadvantage, inseminating fewer females and mating less frequently than nonexposed males (Colinet and Hance 2009; Lacoume et al. 2007; Sutter et al. 2019). Thus, we hypothesized that due to the lasting effects of development at suboptimal temperatures, WA males would be less competitive and less fertile than controls. We also expected fertility potential and size of their reproductive organs to be only partly reversible when WA males are returned to optimal temperatures, as observed in other Drosophila species (Kubrak et al. 2016).

## 2 Material and Methods

### 2.1 Origin and Maintenance of Flies

All experiments were carried out with a mass‐bred population of *D. suzukii*, originally collected in 2016 in Thorigné‐Fouillard, France. From the sampling, 190 isofemale lines were established. They have since been grown and maintained in the laboratory in glass bottles containing around 100 individuals per bottle, filled with a standard food (for 1 L: 30 g brewer yeast, 50 g sucrose, 20 g cornmeal, 15 g agar, 50 g organic carrot powder, and 16 mL of 10% Nipagin). Rearing bottles were stored in a 25°C incubator (MIR‐154‐PE, Panasonic) with a 70% relative humidity and a 12 L/12D light regime. A minimum of 20 bottles was kept every generation. In order to maintain genetic diversity, individuals were shuffled at each generation. To generate flies for the experiments, groups of 15–20 mated females were allowed to lay eggs in 100 mL rearing bottles during a restricted period (< 6 h) under laboratory conditions. This controlled procedure allowed larvae to develop in bottles under uncrowded conditions.

### 2.2 Male Fertility According to Developmental and Adult Temperature

**FIGURE 1:**
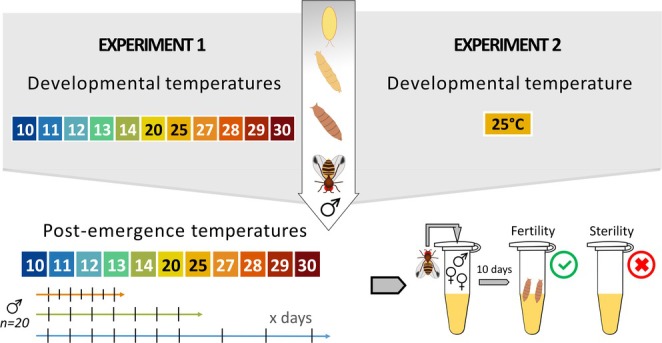
In Experiment 1 (left), eggs were developed into adults under 11 different temperatures, and the fertility of the emerged males was assessed after various durations depending on the temperature. In Experiment 2 (right), eggs were developed into adults at 25°C, and the fertility of the emerged males was assessed under 11 different temperatures and durations. Fertility ratio was determined at each temperature and time point as the proportion of mating tests resulting in progeny (*n* = 20 males per treatment).

We conducted two experiments to assess how developmental and adult temperatures (Experiment 1) and postemergence temperatures (Experiment 2) affected the fertility of males. Thermal fertility thresholds (TFLmin and TFLmax) were evaluated across 11 temperatures, with higher resolution in both extremes: 10°C, 11°C, 12°C, 13°C, 14°C, 20°C, 25°C, 27°C, 28°C, 29°C, and 30°C and 12 L/12D photoperiod (see Figure 1).

In Experiment 1, for each of the 11 developmental temperature conditions, we placed 10 bottles, each filled with 20 mL of media and containing approximately 100 freshly laid eggs. Within 12 h of emergence, 400 males were randomly selected (sexed manually without CO_2_; Colinet and Renault 2012), and groups of 100 males were transferred into bottles containing 10 mL of media. All males were left in their initial developmental temperature after emergence. The timing and frequency of fertility monitoring were adjusted to match the expected pace of reproductive activation at each temperature—slower at cold temperatures and faster at warm ones: daily for 10 days then twice a week for 60 and 65 days at 10°C and 11°C, respectively; daily for 15 days then twice a week for 70 days at 12°C; daily for 20 days then twice a week for 50 days at 13°C and 14°C; twice daily (8 a.m. and 6 p.m.) for 10 days at 20°C, 25°C, 27°C, 28°C, 29°C, and 30°C. Experiments ended when 100% of males were fertile. At 29°C and 30°C, experiments lasted respectively only for 4 and 2 days because of early adult mortality at these stressful temperatures.

In Experiment 2, all males were developed at 25°C before being exposed from emergence to the same 11 different temperatures (see Figure 1). Fertility monitoring followed a similar schedule to that of Experiment 1, with slightly reduced frequency and duration depending on the temperature. At 10°C and 11°C, fertility was assessed twice a week for up to 35 days. At 12°C, we monitored twice a week for up to 45 days. At 13°C and 14°C, monitoring was performed daily for a maximum of 20 days. Finally, at 20°C, 25°C, 27°C, 28°C, 29°C, and 30°C, fertility was monitored twice a day (at 8 a.m. and 6 p.m.) for up to 10 days. Here we did not observe early adult mortality at 29°C and 30°C. Slight differences in timing between experiments reflect observed variations in adult survival, especially at high temperatures.

To assess fertility, each mating trial consisted of placing a treated male with two virgin control females, aged 5 days, reared at 25°C and collected within 6 h of emergence to ensure their virginity and full sexual maturity, in 2 mL Eppendorf tubes containing 1 mL of food (20 replicates at each temperature). The pairs were allowed to mate for 24 h under the male's thermal conditions. After 24 h, the male was removed, and all tubes were placed at 25°C to allow the females to lay eggs and larvae to develop. After 10 days, if one or more pupae had appeared, the male was considered fertile; otherwise, it was considered sterile. Therefore, we considered males to be sterile only if they were alive but did not produce progeny.

The fertility ratio was calculated by determining the number of fertile males out of 20 for each experimental condition (temperature × time postemergence). At each time point, 20 males (*n* = 20) randomly chosen within each experimental condition were tested. After assessing their fertility, these males were discarded (i.e., no repeated measures). This method allowed us to track fertility dynamics over time using independent samples across time, with results expressed as a binomial outcome (fertile vs. sterile) for each time point. The same method was applied to Experiments 1 and 2.

### 2.3 Reproductive Ability of Winter Acclimated (WA) Males

**FIGURE 2:**
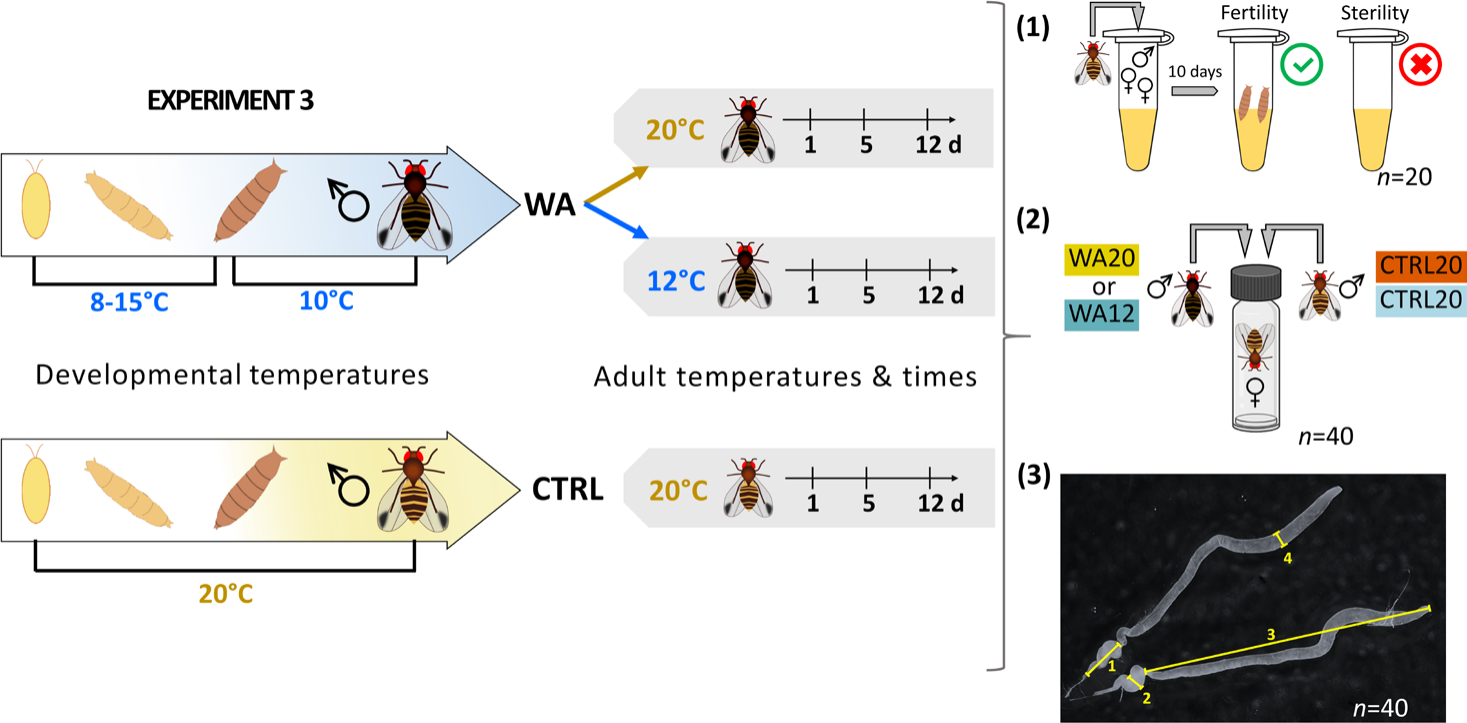
Design of Experiment 3. WA males (dark and cold‐acclimated) were produced by rearing them under low‐temperature conditions. Upon emergence, some WA males were either exposed to 12°C (WA12) or transferred to 20°C (WA20), while control males were reared and maintained at 20°C (CTRL20). For the three groups (WA12, WA20, and CTRL20), three metrics were evaluated at 1, 5, and 12 days postemergence: (1) fertility ratio (*n* = 20), (2) mating competitiveness in choice tests (*n* = 40), and (3) reproductive organ size and color (*n* = 40). The reproductive organ measurements included seminal (1) vesicle length, (2) seminal vesicle width, (3) testis length, and (4) testis width, as shown in the figure.

In the Experiment 3, we created a winter‐acclimated (WA) phenotype to simulate how winter conditions may affect fertility. WA males were produced by developing eggs in bottles under cold alternating temperatures during the larval stage to limit mortality: daily cycle with 8°C for 12 h (8 PM–8 AM) and 15°C for 12 h (8 AM–8 PM). Once the first pupae formed, bottles were placed at 10°C (LD 12/12) until adult emergence. Previous experiments have shown that this protocol results in long development (approximately 50 days) and cold acclimation (Raynaud‐Berton et al. 2024). Moreover, 10°C is close to the fertility threshold (based on methods and results of Experiment 1), so we expected WA males to be under reproductive arrest at emergence. Upon emergence, WA males were separated from females (without CO_2_), and they were divided into two subgroups: one exposed at 12°C to simulate constant chilling after emergence (WA12), and the other was transferred to 20°C (WA20) to simulate end‐of‐winter conditions and assess recovery of fertility at higher temperature. As control, we developed and left males at 20°C (CTRL20). For the three groups (WA12, WA20 and CTRL20), we evaluated three metrics after 1, 5, and 12 days postemergence: fertility ratio, mating competitiveness, and reproductive organs size and color (see Figure 2).

1. For fertility, the same procedure as in Experiments 1 and 2 was repeated, with again 20 replicates (20 random males) per experimental condition (WA12, WA20, CTRL20 × 3 time points).
2. For mating competitiveness, choice tests were conducted, with one WA male competing against one CTRL male that developed at 20°C. The two competitors were placed inside a glass tube together with a virgin control female (reared at 20°C). To maintain the males' conditions (12°C or 20°C) during the assays, copulation behaviors were assessed by immersing the glass tubes in an aquarium regulated either at 20 or 12°C for WA20 and WA12 males, respectively. At 20°C, we assessed WA20 versus CTRL20 males, after 1, 5, and 12 days post emergence, and at 12°C, we assessed WA12 versus CTRL20 males, after 1, 5, and 12 days post emergence. The water in the tank was regulated using an immersion heater (Variostat CC, Pilot One, Huber). Mating tests were conducted in the morning (8 AM) and lasted for 3 h. 40 mating tests were performed per experimental condition (*n* = 40). When copulation occurred, the “winner” was identified; this was facilitated by the visibly darker coloration of WA males (see Colinet and Kustre 2025). We also performed mating assays with two control males reared and maintained at 20°C (CTRL20) at days 1, 5, and 12.
3. To measure the size of the reproductive organs, males (WA12, WA20, CTRL20) were dissected in phosphate‐buffered saline (PBS) under a microscope (Zeiss Stemi 2000‐C). Pictures were taken with a Zeiss AxioCam ERc5s camera using Zen lite 3.1 Blue Edition software. Dimensions of reproductive organs ((1) seminal vesicles length, (2) seminal vesicles width, (3) testes length, and (4) testes width, see Figure 2) were measured with the Zen software after calibration, with again 40 males dissected (*n* = 40) per experimental condition (WA12, WA20, CTRL20 × 3 time points). The width of the testis and seminal vesicle was measured at their distal and midsections, respectively, corresponding to the most consistent and widest parts of the structures. These locations were selected based on visual anatomical landmarks and applied systematically to all images. To indirectly estimate reproductive maturity, during dissections we also observed and categorized the coloration of the testes and seminal vesicles into four qualitative levels, ranging from white (level #1) to intense yellow (level #4) (see Figure 6). This method served as a proxy for the accumulation of mature sperm and seminal fluids, with yellowish coloration indicating a higher degree of reproductive maturity. The yellow coloration as an indicator is based on previous studies showing that pigmentation in the testes of *Drosophila* species is linked development of pigment cells, signaling sexual maturity and fertility as well as overall reproductive readiness (Morris et al. 2009; Olivera Crego 2017; Papagiannouli and Lohmann 2012; Yan et al. 2020). Although this measure is qualitative, it provides an indirect estimate of reproductive maturity, complementing the mating trials and morphological data.

### 2.4 Statistical Analyses

All statistical analyses were performed in RStudio (R Core Team 2023). The fertility ratio was analyzed using generalized linear models (GLM) with a binomial distribution (i.e., sterile vs. fertile) and a logistic link function to examine the effects of temperature, time postemergence, and their interaction. A complete separation may occur in logistic/binomial regression when some categories contained 0% or 100% success (or failure). To account for this situation that occurred in some conditions, a bias reduction method was implemented using *brglmFit* as the fitting method for GLMs within the *brglm2* package (Kosmidis et al. 2017). To compare the temporal dynamics of fertility variation at each temperature, we extracted the coefficients (log odds) from each individual logistic model. These coefficients represent the change in the log odds of fertility for a one‐unit change in the predictor variable (i.e., time), analogous to the slopes in linear regression models. By plotting these coefficients, we were able to visualize the rate of change in fertility over time at each temperature. To model the thermal reaction norms for fertility and estimate the thermal fertility limits (i.e., TFLmin and TFLmax), we used the fertility ratio estimated at 10 days postemergence, as this duration was common in all experimental conditions. We applied the *rTPC* and *nls.multstart* packages to model the fertility as a function of temperature (T) (Padfield et al. 2021). Among the nonlinear models proposed in *rTPC*, we selected the best equation based on the lowest AIC, the relevance of estimated parameters, and the quality of data fit.

For Experiment 1, the best equation was the model “Ratkowsky” (Padfield et al. 2021):

$$\text{Ratio}\;\left(T\right)={\left(a\cdot \left(T-T_{\min}\right)\right)}^{2}\cdot {\left(1-\exp\left(b\cdot \left(T-T_{\max}\right)\right)\right)}^{2}$$

For Experiment 2, the best equation was the model “Thomas 2” (Padfield et al. 2021):

$$\text{Ratio}\;\left(T\right)=a.\exp^{b.T}-\left(c+d.\exp^{e.T}\right)$$

For the third experiment, the effect of the predictors (treatment and time) on the fertility ratio was also analyzed with a GLM logit together with a bias reduction method implemented using *brglmFit*. Mating success in the choice tests was analyzed using Fisher's exact tests to compare the proportions of successful matings between WA and CTRL males, at 20°C and 12°C separately. For anatomical comparisons, we analyzed the effects of treatment and time on each dependent variable (i.e., testis length, testis width, seminal vesicle length, and seminal vesicle width) using 2‐ways ANOVAs followed by post hoc tests in *emmeans* R package. To assess whether the distribution of color categories differed between treatments (WA12, WA20, CTRL20) at the different time points (1, 5, and 12 days), we performed exact Fisher's tests for each time point separately.

## 3 Results

### 3.1 Males Fertility Based on Combined Developmental and Adult Temperature (Experiment 1)

**FIGURE 3:**
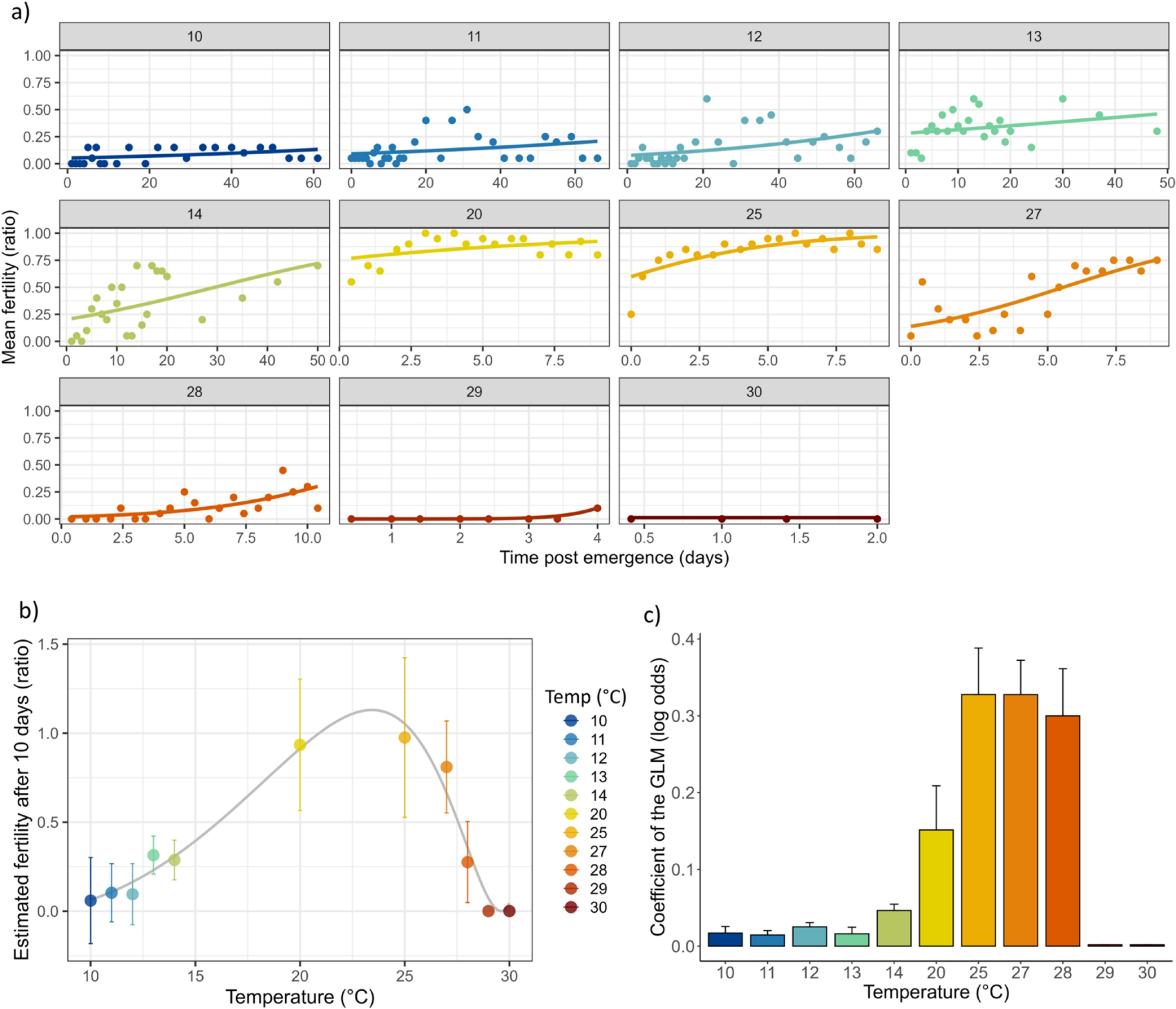
Experiment 1: (a) Change in fertility with time postemergence at each developmental and adult temperature from 10°C to 30°C as indicated within each graph. The lines represent the predictions of GLM logit models. (b) Fertility ratio at 10 days postemergence according to developmental temperature (±SE). The line shows the TPC for fertility modeled with Ratkowsky nonlinear equation. (c) Log odds coefficients (±SE) obtained from the logit models and representing the temporal dynamics of fertility change for each temperature.

Results from the GLM revealed significant effects of all factors. Time postemergence (i.e., age of males) had a strong positive effect on fertility ratio (Chi^2^ = 80.27; Df = 1; *p* < 2.2e^−16^), indicating that fertility increased with time, as males matured. Temperature also significantly affected fertility (Chi^2^ = 1622.48; Df = 10; *p* < 2.2e^−16^), and a significant interaction between time and temperature was observed (Chi^2^ = 131.01; Df = 10; *p* < 2.2e^−16^). To account for this interaction, we also analyzed each temperature separately using logit models. Between 10°C and 29°C, time postemergence had a significant effect on fertility (Df = 1; *p* < 0.001; Figure 3a), but at 30°C, no significant effect of time was detected due to complete sterility and early mortality (Figure 3a). At 29°C, only two males were fertile on day 4. The coefficients of the logistic regressions (log odds) were plotted in Figure 3c to visualize the rate of change in fertility over time at each temperature. The values ranged from 0.002 to 0.33 (Figure 3c). This range suggests that the impact of time postemergence on fertility was generally small at lower temperatures (< 14°C), with values being minimal at cold, and larger at higher temperatures (20°C–28°C). No accurate estimation was possible at 29°C and 30°C due to sterility and early adult mortality. Next, we modeled the estimated fertility at 10 days according to developmental conditions and obtained a nonlinear TPC for fertility, as well as estimates for TFLs (Figure 3b). The outputs of the Ratkowsky model were: achieved convergence = 1.49e^−08^, AIC = −17.35, Topt = 23.4°C, TFLmin = 9.8°C, TFLmax = 29.0°C. TFL20_cold and TFL20_heat (i.e., the temperatures at which fertility dropped to 20% below and above Topt, equivalent to 80% sterility; see Parratt et al. 2021) were estimated to be 12.6°C and 28.5°C, respectively.

### 3.2 Males Fertility Based on Adult Temperature (Experiment 2)

**FIGURE 4:**
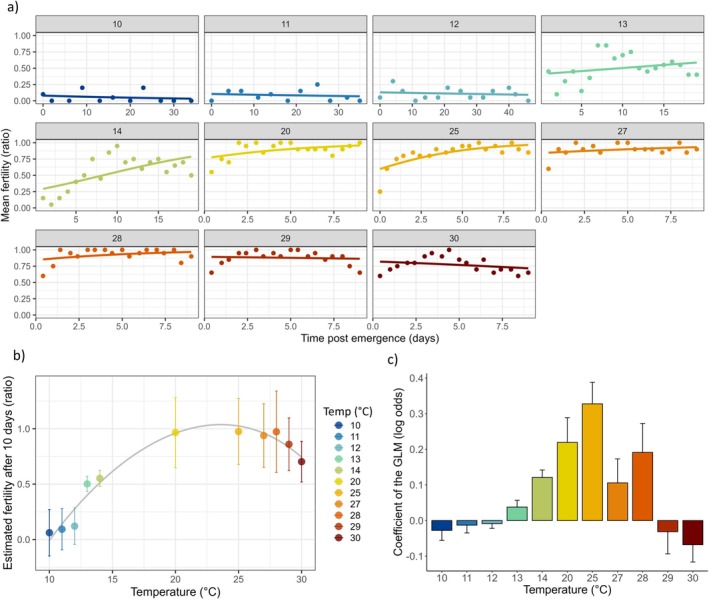
Experiment 2: (a) Change in fertility ratio with time postemergence at each adult temperature from 10°C to 30°C as indicated within each graph. The lines represent the predictions of GLM logit models. (b) Fertility ratio at 10 days postemergence according to adult temperature (±SE). The line shows the TPC for fertility modeled with Thomas 2 nonlinear equation. (c) Log odds coefficients (±SE) obtained from the logit models and representing the temporal dynamics of fertility change for each adult temperature.

At sub‐ and supraoptimal adult temperatures (10°C, 11°C, 12°C and 29°C, 30°C, respectively), the fertility ratio remained very low and did not show signs of progression over time (Figure 4a). In some cases, a temporal decline in fertility was observed (e.g., at 10°C, 29°C, or 30°C; see Figure 4a). Results of the analysis showed that the time postemergence had a significant effect on the fertility ratio (Chi^2^ = 16.06; Df = 1; *p* = 6.12e^−05^). We also observed a significant effect of adult temperature (Chi^2^ = 1106.64; Df = 10; *p* < 2.2e^−16^) and an interaction between time postemergence (i.e., age of males) and temperature (Chi^2^ = 85.75; Df = 10; *p* = 3.71e^−14^). The pace of temporal fertility changes at each temperature (i.e., logg odds) had very low values and remained rather stable (or even negative) at temperatures below 13°C (Figure 4c). Between 13 and 28°C, the fertility ratio gradually increased with time, resulting in higher and positive log odds values. At supra‐optimal temperatures (29°C and 30°C), the fertility ratio decreased with time, resulting in negative log odds values (Figure 4a,c). Finally, we modeled the estimated fertility at 10 days postemergence according to adult temperature (Figure 4b) and the outputs of the Thomas2 model were: achieved convergence = 1.49e^−08^, AIC = −15.4, Topt = 23.6°C, TFLmin = 10.05°C, TFLmax = 34.8°C. TFL20_cold and TFL20_heat (i.e., the temperatures at which fertility dropped to 20% below and above Topt, see Parratt et al. 2021) were estimated to be 11.4°C and 33.9°C, respectively. In this case, TFL20_heat should be considered with caution, as it is an extrapolation beyond the temperatures observed in the experimental data.

### 3.3 Reproductive Ability of Winter‐Acclimated Males (Experiment 3)

**FIGURE 5:**
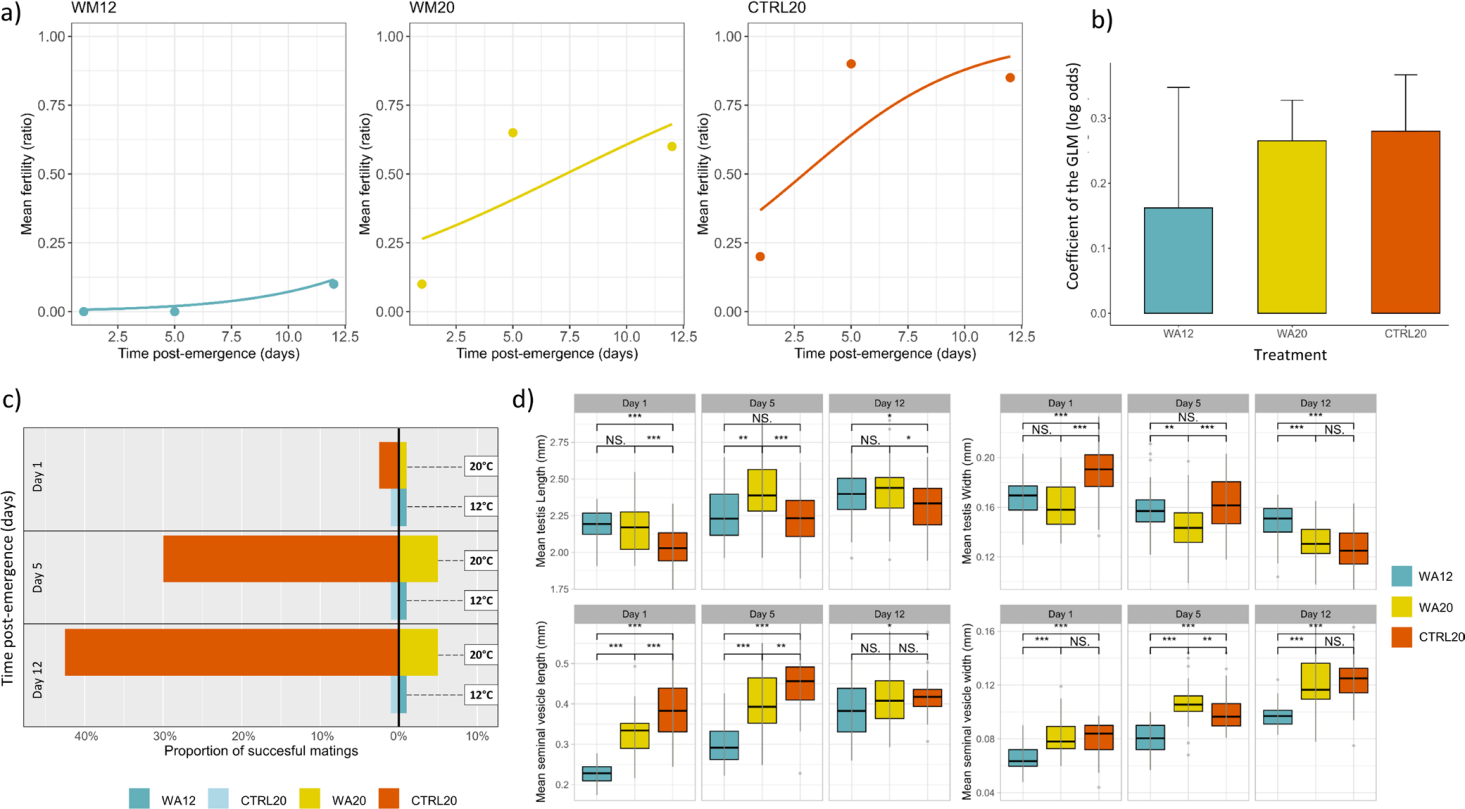
(a) Changes in fertility ratio according to time after emergence in the three different treatment groups: WA12, WA20, and CTRL20. The lines represent the predictions from GLM logit models. (b) Log odds coefficients obtained from each GLM logit model (± SE), illustrating the pace of temporal fertility changes for each group. (c) Proportion of successful mating out of 40 trials, after 1, 5 and 12 days postemergence and when assessd either at 20°C (WA20 vs. CTRL20) or at 12°C (WA12 vs. CTRL20). (d) Boxplots showing mean size of males reproductive organs at three different time points: Day 1, 5, and 12 postemergence, and for the three different treatment groups: WA12, WA20, and CTRL20. * *p* < 0.05;** *p* < 0.01; *** *p* < 0.001; NS = not significant.

In this experiment, we compared male fertility across three thermal treatments (WA12, WA20, and CTRL20) and three different time points after emergence (1, 5, and 12 days). The fertility ratio was rather low on day 1 for all treatments (Figure 5a). When WA males were left at 12°C (WA12), fertility remained close to zero, with only 2 out of 20 males becoming fertile after 12 days. When WA males were returned to 20°C at emergence (WA20), fertility recovered quickly, reaching 60% after 12 days. Control males (CTRL20) at 20°C showed a similar pattern as WA20, but fertility reached up to 90% (Figure 5a). Overall, time postemergence had a significant effect on the fertility ratio (Chi^2^ = 26.59; Df = 1; *p* = 2.50e^−07^), as did the thermal treatment (Chi^2^ = 69.39; Df = 2; *p* = 8.53e^−16^), but there was no significant interaction between time and treatment (Chi^2^ = 1.43; Df = 2; *p* = 0.489). Post hoc tests showed that on day 1, no significant differences were found among treatments (*p* > 0.05 in all comparisons). On day 5, fertility was significantly lower in WA12 compared to both CTRL20 and WA20 (*p* < 0.05), but no significant difference was observed between CTRL20 and WA20 (*p* > 0.05). On day 12, fertility was again significantly lower in WA12 compared to CTRL20 and WA20 (*p* < 0.05), with no significant difference between CTRL20 and WA20 (*p* > 0.05). The log odds of the models depicted a faster fertility progression with time in WA20 and CTRL20 treatments than in WA12 (Figure 5b).

We compared the success of mating in competition tests between CTRL and WA males (Figure 5c). At 12°C, no mating was observed in any of the trials (neither in WA12 nor in CTRL20). At 20°C, on day 1, there was only one successful mating with a CTRL20 male out of 40 trials. On day 5 at 20°C, a significant difference was found in the competition between CTRL20 and WA20, with only one successful mating for WA20 compared to 12 successful matings for CTRL20 (Fisher test: *p* = 0.0014). By day 12 at 20°C, the difference became even more pronounced, with only two successful matings for WA20 compared to 17 matings for CTRL20 (Fisher test: *p* = 0.0001) (Figure 5c). In the controls with two CTRL20 males competing for the virgin female, there were 2, 25, and 21 successful matings out of 40 trials on days 1, 5, and 12 (i.e., 5%, 62%, and 52% success respectively).

Testis length increased significantly with time (Chi^2^ = 83.41; Df = 1; *p* < 2.2e^−16^). It varied with treatments (Chi^2^ = 35.21; Df = 2; *p* = 2.26e^−08^) and there was no interaction (Chi^2^ = 0.293; Df = 2; *p* = 0.863). Testis width significantly decreased over time (Chi^2^ = 208.43; Df = 1; *p* < 2.2e^−16^). It varied with treatments (Chi^2^ = 33.57; Df = 2; *p* = 5.11e^−08^) and there was an interaction (Chi^2^ = 59.07; Df = 2; *p* = 1.48e^−13^). The seminal vesicle length showed a gradual increase over time (Chi^2^ = 102.77; Df = 1; *p* < 2.2e^−16^). It varied with treatments (Chi^2^ = 194.81; Df = 2; *p* < 2.2e^−16^) and there was an interaction (Chi^2^ = 46.40; Df = 2; *p* = 8.36e^−11^). The seminal vesicle width also increased significantly over time (Chi^2^ = 356.22; Df = 1; *p* < 2.2e^−16^) and it varied with treatments (Chi^2^ = 158.40; Df = 2; *p* < 2.2e^−16^) and there was an interaction (Chi^2^ = 6.31; Df = 2; *p* = 0.042). While at day 1 all traits were different between WA males and CTRL20, the multiple comparisons showed that after 12 days, traits measured on reproductive organs were similar between WA20 and CTRL20 males (testis width, seminal vesicle length and width, *p* > 0.05), while all the traits were different between WA12 and CTRL20 males (*p* < 0.05) (Figure 5d).

**FIGURE 6:**
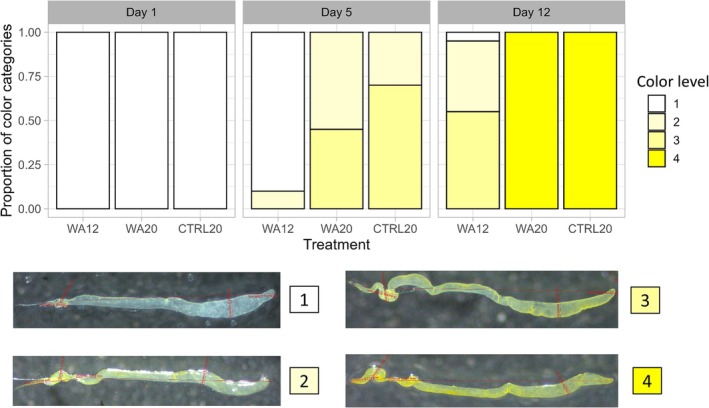
Bar plots show the proportion of seminal vesicles and testes in each of the four color categories (level #1 to #4) at Day 1, Day 5, and Day 12 postemergence for three experimental groups: WA12, WA20, and CTRL20. Representative photos at the bottom illustrate the four color levels: #1 (colorless), #2 (light yellow), #3 (yellowish), and #4 (intense yellow).

About the color of reproductive organs (Figure 6), at Day 1, all males (WA12, WA20, CTRL20) exhibited colorless seminal vesicles and testes (level #1), indicating no visible signs of reproductive maturation.

By Day 5, the seminal vesicles and testes of WA20 and CTRL20 males began to show yellowish coloration (levels #2 and #3), while WA12 males remained mostly colorless (level #1).

At Day 12, all WA20 and CTRL20 males had intense yellow seminal vesicles and testes (level #4), whereas WA12 males remained in a lighter yellowish state (levels #2 and #3) or even colorless (level #1). The distribution of color categories was significantly different between the treatment groups (WA12, WA20, CTRL20) at Day 5 and 12. At Day 5, the analyses revealed a significant difference (Fisher test: *p* = 6.75e^−27^) and at Day 12, the difference was even more pronounced (Fisher test: *p* = 2.62e^−32^). No significant difference was observed at Day 1 (*p* = NA), as all males displayed clear, colorless seminal vesicles and testes (level #1), resulting in uniform categorization across the groups.

## 4 Discussion

In this study, we examined how various thermal conditions during the development and at the adult stage affected males' fertility in *D. suzukii*. We also explored the reproductive ability of males reared under winter‐like conditions. The fertility of *D. suzukii* males exhibited typical thermal performance curve (TPC) patterns with both developmental and adult temperatures, showing a gradual increase in fertility with temperature up to an optimum zone, followed by a decline at higher temperatures (Araripe et al. 2004; David et al. 2005; Wang and Gunderson 2022). As expected, the TPC for fertility was narrower than that for viability. For developmental temperatures, the model estimated the FTLmin and FTLmax at 9.8°C and 29.0°C, respectively. In comparison, Raynaud‐Berton et al. (2024) reported Tmin and Tmax for viability at 8°C and 31°C, while Colinet and Kustre (2025) found these values to be 7.6°C and 30.3°C, respectively. We thus confirm that TFLs exhibit a narrower tolerance range compared to survival, with values just a few degrees higher and lower than the cold and heat limits for survival (Ørsted et al. 2024; Parratt et al. 2021; Van Heerwaarden and Sgrò 2021; Walsh et al. 2019). The value for cold‐induced sterility threshold falls within the range values observed in other *Drosophila* species, typically between 10°C and 14°C, depending on the species (Araripe et al. 2004; Chakir et al. 2002; Mensch et al. 2016; Kreiman et al. 2023).

Our results showed a significant effect of time postemergence on fertility ratio across all developmental temperatures. Fertility ratio continued to rise as adults aged, except at 29°C and 30°C, where the adult survival and consequently the reproduction was limited to only 4 and 2 days, respectively. This aligns with existing literature, which indicates that adult survival is very short at these stressful constant temperatures (Enriquez and Colinet 2017; Raynaud‐Berton et al. 2024; Tochen et al. 2014). Log odds from models based on developmental temperatures revealed positive coefficients, indicating an increased probability of male fertility with time. These were very low between 10°C and 13°C, but increased sharply between 14°C and 28°C. At 29°C and 30°C, however, the values dropped to near zero, highlighting the high susceptibility of males that developed and remained at temperatures above 28°C. Our data are also coherent with a previous study showing that the fertility of adult *D. suzukii* was drastically reduced when development occurred at 28°C (Faria et al. 2023) and null when males developed at 30°C (Green et al. 2019). These authors reported that chronic heat exposure during development caused an inability to load sperm into the seminal vesicle (Green et al. 2019). A similar effect was observed by Porcelli et al. (2017) in *Drosophila subobscura* males, where high temperatures during development led to a significant reduction in motile sperm in the seminal vesicles.

An important finding was that the combined effects of supra‐optimal temperatures (≥ TFLmax) during both development and at the adult stage (Experiment 1) appeared far more detrimental to both survival and reproduction than when supra‐optimal temperatures were experienced only after emergence (Experiment 2). In Experiment 2, males remained alive for at least 10 days but exhibited a progressive decline in fertility, despite fertility levels remaining quite high. In contrast, in Experiment 1, at 29°C and 30°C, emerged males were sterile and also showed early mortality. This suggests that chronic exposure to supra‐optimal temperatures throughout development has severe and lasting consequences, which seem much more detrimental than exposures only at the adult stage. In *D. melanogaster* , when short‐term heat stress was applied during early developmental stages (egg, L1, L2, and L3), it did not result in a reduction in male fertility. In contrast, males chronically exposed to 29°C–30°C throughout the entire developmental cycle exhibited low fertility and were unable to fully recover (Canal Domenech and Fricke 2023; Rohmer et al. 2004; Zwoinska et al. 2020). Similar findings were reported in *Drosophila subobscura* (Simoes et al. 2020). Cumulative exposure up to the pupal stage appears particularly detrimental to male fertility, probably because the final steps of spermatogenesis during the pupal stage are particularly sensitive to heat stress (Canal Domenech and Fricke 2023). Altogether, these findings suggest that chronic heat exposure during development has different outcomes on fertility than exposure during the adult stage.

We found that males exposed to developmental temperatures below 13°C–14°C were not entirely sterile and could actually progressively become fertile, albeit very slowly, if maintained at these low temperatures for several weeks (Experiment 1). However, below 9.8°C (TFLmin), fertility may be severely compromised. Males developing and emerging after winter could experience a similar situation, where spermatogenesis is drastically slowed, akin to the reproductive dormancy (quiescence) in females, in which oogenesis is deeply slowed below 14°C (see Colinet and Kustre 2025). This resembles a reproductive dormancy‐like syndrome in males, likely due to the impact of low temperature on spermatogenesis during both development and the adult stage, as reported in other *Drosophila* species (Kubrak et al. 2016; Kreiman et al. 2023). Grassi et al. (2018) dissected *D. suzukii* males captured in the field throughout the year and found that, on average, less than 40% of males contained sperm during the winter months. This suggests that, similar to females, male populations are largely in a temperature‐induced reproductive dormancy during this period. From ecological, demographic, and agricultural perspectives, these findings are relevant in the context of climate change. Warmer winters may shorten the dormancy‐like period in males, resulting in a higher number of fertile males available to females in the early season, which could significantly impact population dynamics as well as pest control (Tait et al. 2021). While reproductive dormancy in females is well defined by immature previtellogenic ovaries (Kubrak et al. 2014; Lirakis et al. 2018), in a next step, the precise diagnosis of reproductive dormancy in males will need to be further characterized.

In holometabolous insects, pupal and early adult stages are often more sensitive to thermal induced‐sterility than earlier developmental stages such as larvae (Canal Domenech and Fricke 2023; Meena, Maggu, et al. 2024; Sales et al. 2021; Wang and Gunderson 2022), possibly due to a reduced ability to repair damage and remodel morphology during spermatogenesis and metamorphosis (Meena, De Nardo, et al. 2024). In the second experiment, we tested the fertility of *D. suzukii* males that had all developed at 25°C and that were then exposed to chronic exposures to different temperatures after emergence for varying durations. As hypothesized, we found that exposing males during sexual maturation to temperatures outside the optimal range (i.e., below 13°C and above 28°C) reduced their fertility. Fertility ratio increased gradually with postemergence time and temperature until reaching an optimal range between 20°C and 28°C, after which it decreased at temperatures above 28°C. The estimated thermal limits from the adult TPC were TFLmin: 10.05°C and TFLmax: 34.8°C. The TFLmin value is close to the one observed in Experiment 1. The log odds revealed negative trends at low temperatures (below 13°C), suggesting that males developed at 25°C but kept at low temperatures could not reach full sexual maturation and may even progressively become sterile with chronic adult cold exposure. This is consistent with other observations in different insects where cold‐exposed males had a reduced sperm stock compared to control males, leading to lower fertility and mating success (e.g., Kvelland 1965; Kubrak et al. 2016; Lacoume et al. 2007). Regarding TFLmax, the estimated value of 34.8°C is notably higher than that obtained from developmental temperatures. Thus, it seems that adult stages are not more sensitive than earlier developmental stages (e.g., pupal), as previously reported (Canal Domenech and Fricke 2023; Sales et al. 2021). In *D. suzukii*, TFLmax (or TFL80) was reported to be around 33°C when a short‐term stress was applied at the adult stage (Parratt et al. 2021). It is important to distinguish between acute (intense but short‐term) and chronic effects (prolonged exposure), as the latter may lead to progressive sterility at temperatures much lower than with acute stress. For example, in *D. melanogaster*, a TFL80 close to 36°C was reported after 4 h of exposure (Parratt et al. 2021), but sterility can occur at lower temperatures, for instance when males are maintained at 29°C for 20 days (Gandara and Drummond‐Barbosa 2023). Our data corroborates these earlier observations. In the field, it is likely that exposures involve both chronic and acute stress, but the insects can probably also benefit from periods of recovery (Colinet et al. 2015) and/or take refuge in buffered microhabitats (Pincebourde and Woods 2020). We also noted that above 28°C, initially fertile males gradually became sterile, suggesting that with prolonged exposure (longer than 10 days), adults could eventually have become fully sterile, as observed in *D. melanogaster* males maintained at 29°C for 20 days (Gandara and Drummond‐Barbosa 2023). Therefore, in addition to emphasizing that TFLs depend on life stage exposure, our data also highlight that males' reproductive capacity also depends on the intensity of stress, as well as the duration of exposure. Recent studies have shown that, like critical thermal limits (CTmin and CTmax), key life history traits such as reproduction follow a robust exponential relationship between exposure time and temperature (e.g., Ørsted et al. 2024), meaning that the now well‐established TDT approach (i.e., Thermal Death Time) (Jørgensen et al. 2022; Ørsted et al. 2022; Rezende et al. 2020) also applies to reproductive traits. This implies that the studies identifying sublethal effects of temperature on fertility have largely overlooked the crucial interaction between exposure intensity and duration (e.g., Canal Domenech and Fricke 2023; Green et al. 2019; Parratt et al. 2021; Sales et al. 2021; Van Heerwaarden and Sgrò 2021; Zwoinska et al. 2020). Future studies could explore longer exposure periods to determine the moment at which males become fully sterile at a range of stressful temperatures.

While male sterility at high temperatures has been extensively studied in *Drosophila* (Canal Domenech and Fricke 2022, 2023; Gandara and Drummond‐Barbosa 2022; Green et al. 2019; Parratt et al. 2021; Porcelli et al. 2017; Rohmer et al. 2004), male sterility at low temperatures remains largely uncharacterized (but see Kvelland 1965 and Kubrak et al. 2016). To our knowledge, this study is the first to broadly investigate chronic cold‐induced sterility in *D. suzukii*. Similar to high temperature, prolonged low temperature was reported to induce male sterility in cactophilic species (Kreiman et al. 2023). Our data show that exposing males during sexual maturation to suboptimal temperatures (below 13°C) prevented and reduced their fertility, maybe due to a decline in sperm production and viability. In females, previous data have shown that chronic exposure of adult *D. melanogaster* to warm or cold temperatures leads to a significant reduction in egg production, but through completely distinct mechanisms between heat and cold (Gandara and Drummond‐Barbosa 2022). Similarly, chronic exposure to sub‐ and supraoptimal temperatures may affect sperm production in *D. suzukii* males through mechanisms that may be distinct and that remain to be characterized.

Our method to assess sterility, based on the presence of pupae in progeny, may not capture instances where larvae were present but did not reach the pupal stage. Such an outcome would suggest that temperature stress indirectly affected progeny survival (i.e., larval survival) rather than male fertility itself, as neither the females nor the progeny were exposed to stress (i.e., kept at 25°C). This effect might arise through paternal contributions to progeny survival, potentially linked to stress‐induced changes in sperm or other paternal factors influencing offspring development. From a fitness perspective, this could be considered a form of sterility, as no viable offspring would be produced by stress‐exposed males.

Body size variation may have influenced fertility, but our results suggest that thermal exposure during both development and adulthood is likely the primary driver of the observed fertility patterns. In Experiment 1, where development occurred under different temperatures, we would have expected body size to be greater at low temperatures, and thus fertility to be higher in cold‐developed males. Yet, we observed a clear TPC with an optimal fertility range around 23°C, which does not correspond to maximal body size (Colinet and Kustre 2025). Despite potential size differences, both experiments revealed consistent TPCs with an optimal fertility range around 23°C, suggesting that temperature, rather than body size, primarily shaped fertility.

In the last experiment, we aimed to assess the reproductive capacity of WA males to decipher their ability to mate and recover fertility after prolonged cold exposure, as it may occur at the end of winter. Our findings showed that fertility significantly increased over time in all treatments, yet the control group (CTRL20) exhibited the highest fertility ratio. Comparing fertility at different time points (days 1, 5, and 12 postemergence) allowed to decipher the temporal dynamics of fertility in the three experimental groups. We observed low fertility and no differences among the groups on day 1, likely due to incomplete sexual maturation on the first day postemergence (Perrin‐Waldemer 1966). However, by days 5 and 12, CTRL20 and WA20 males exhibited similar fertility ratios, while WA12 males remained largely infertile. This confirms our hypothesis that WA males can rapidly regain fertility when returned to optimal conditions. Panel et al. (2020) reported that *D. suzukii* males developed at low temperatures and then exposed to a prolonged cold period were mostly sterile. This scenario corresponds to the males exposed to low temperatures in our Experiment 1 or to the WA12 males in Experiment 3, and our data corroborate this observation. However, our findings also show that when these cold‐developed males (i.e., winter acclimated phenotype) are returned to optimal temperatures, they can quickly regain fertility, indicating that chronic mild cold does not permanently affect fertility in *D. suzukii* males. This suggests that after winter, males in the field will still be capable of inseminating females once temperatures become favorable.

We hypothesized that due to the lasting effects of development at suboptimal temperatures, WA males might be less competitive and less fertile than controls. Behavioral experiments showed no mating activity at 12°C, suggesting that this temperature was too cold to induce mating even in WA males, as seen in other species (Meats and Fay 2000). At 20°C, some mating activity was recorded in both the WA20 and CTRL20 groups on days 5 and 12. However, we found a relatively low competitiveness of WA20 males compared to CTRL20 males, supporting our hypothesis that development under winter‐like cold conditions may induce long‐lasting effects on male reproductive performance. This result aligns with studies in various species showing that males exposed to thermal stress are at a disadvantage, mating less frequently and inseminating fewer females than unexposed males (Colinet and Hance 2009; Kubrak et al. 2016; Lacoume et al. 2007; Sutter et al. 2019). In *D. melanogaster* , Kubrak et al. (2016) also showed that even after 3 weeks of recovery from cold‐induced dormancy, when the male reproductive organs were fully mature, the percentage of copulation remained very low in males that had experienced dormancy compared to control males. In other *Drosophila* species, acclimation under chronic winter‐like temperatures also negatively affected courtship duration and mating success, due to altered courtship song, as reported in *Drosophila littoralis* and *Drosophila montana* (Aspi and Hoikkala 1995; Hoikkala and Isoherranen 1997). Developmental acclimation markedly improves cold survival (Enriquez et al. 2018; Enriquez and Colinet 2019), but this may come at a cost (trade‐off) on reproductive potential (Everman et al. 2018). The reduced attractiveness of WA males could be due to several factors, including morphological changes, such as increased body size, wing length, or darker coloration of this phenotype (Colinet and Kustre 2025). Additionally, courtship success in many *Drosophila* species is linked to traits like locomotor activity, running speed, and performance in aggressive interactions with rival males (Partridge et al. 1985; Sisodia and Singh 2009), which could be impaired by low developmental temperatures (Enriquez et al. 2018; Grandela et al. 2024). Interestingly, a recent study in *D. suzukii* found that winter‐like phenotypes (developed at 12°C) had distinct cuticular hydrocarbon (CHC) profiles compared to controls (Kárpáti et al. 2023). These changes in CHC profiles may enhance survival under cold conditions and prevent desiccation, but they could also impact mate recognition cues. In fact, Kárpáti et al. (2023) reported that WA males exhibited reduced mating success and shorter mating durations. Our data thus support the idea that adaptations for winter survival may come at the cost of reduced mating competitiveness. While WA males appeared to recover fertility quickly, their sexual attraction seemed negatively affected by several putative factors, including altered morphology, courtship behavior, and CHC profiles.

We compared anatomical traits between WA12, WA20, and CTRL20 males, focusing on testis and seminal vesicle sizes as proxies for fertility. Across all treatments, testis length and seminal vesicle size (both length and width) increased over time, while testis width decreased. We used testis size as a proxy for fertility because it is directly related to sperm production, and its growth is indicative of reproductive maturation. As described by Miller (1941), in freshly emerged teneral *Drosophila* males, the testes are turgid, thick, and proportionally shorter, with less of their length extending freely from the coil. Over time, as the males mature, the testes elongate and become thinner, reflecting the ongoing maturation process and sperm production (Miller 1941; Pitnick 1996), as observed in CTRL20 males. Testis size was thus considered here as an indicator of male fertility (like the yellow color), as it correlates with sperm production capacity, which is critical for successful mating and fertilization. In *D. suzukii*, testis length has been shown to increase with time of maturation (Faria et al. 2023), and similar trends in seminal vesicle size with maturation time have been observed in *D. melanogaster* (Kubrak et al. 2016). However, we recognize a limitation in our study in that we did not correct our measurements of testis and seminal vesicle sizes for body size. That said, we believe this to be of limited concern for the following reasons. A simple allometric effect is unlikely to account for the observed differences. If a typical temperature–size relationship were the primary driver, we would expect WA12 and WA20 males (developed under cold conditions) to exhibit larger reproductive organs than CTRL males at emergence. However, our data did not support this trend; in fact, we found the opposite: CTRL males had larger reproductive metrics than WA males in three out of four traits at day 1 postemergence. This suggests that the observed differences are not simply due to variation in body size but are more likely a result of cold‐induced impairment in reproductive organ development and/or delayed maturation in WA males at cold.

On day 1 after emergence, all traits were significantly different between WA males and CTRL20 males. However, by day 12 postemergence, the reproductive organ traits (testis width, seminal vesicle length and width) were similar between WA20 and CTRL20 males, while all traits remained different between WA12 and CTRL20 males. These observations suggest that the reproductive organs of WA20 males returned to a morphological state and likely function comparable to the control at optimal temperatures. Regarding seminal vesicle width, an indicator of sperm production and storage (Perrin‐Waldemer 1966), our results suggest that WA males placed at 20°C postemergence were capable of developing sperm at a comparable rate to CTRL males, whereas those kept at 12°C showed limited sperm accumulation. Even though cold‐exposed males (WA12) initially showed reduced fertility due to limited sperm production (as indicated by smaller seminal vesicle sizes), these males were capable of recovering reproductive function when returned to optimal temperatures (e.g., 20°C). This suggests that low temperatures during development or early postemergence induce temporary infertility, but they do not necessarily lead to irreversible reproductive arrest or damage. Similarly, in *D. melanogaster* males that had recovered for 1–3 weeks after 3 weeks of dormancy, the seminal vesicles and accessory glands progressively increased in size, the latter eventually reaching the size of controls (Kubrak et al. 2016).

The observed color changes in the testes and seminal vesicles across the experimental groups support our interpretation of differential reproductive maturity. The yellowing of these organs in *Drosophila* males is a well‐established indicator of sexual maturation and fertility (Morris et al. 2009; Papagiannouli and Lohmann 2012). While sperm presence was not directly assessed, the color intensity can serve as a proxy for reproductive function, with yellowing correlating with the development of pigment cells in the testes (Morris et al. 2009; Papagiannouli and Lohmann 2012). This pigmentation also functions as a visual cue for females, signaling male readiness to mate (Olivera Crego 2017). Our findings also align with previous studies on *D. suzukii*, which demonstrated that the absence of yellow pigmentation was associated with mating failure and functional limitations of the testes (Yan et al. 2020). The absence or significant delay of yellowing in WA12 males further supports the conclusion that they remained reproductively immature, while WA20 showed signs of fertility recovery.

Our findings suggest that the reproductive organs of WA males, after being returned to an optimal temperature, recovered both in terms of morphological dimensions and function, as also confirmed by fertility assays. Kubrak et al. (2016) reported that the male reproductive system requires a longer recovery time after dormancy to resume function than females. Here, we confirm that recovery from cold‐induced infertility indeed required some days (i.e., 12 days). This is significant for understanding the reproductive potential of fly populations after winter. Males that have experienced low temperatures may be initially less reproductively active, but as temperatures rise, they could regain normal reproductive function. While low temperature during and after development results in reproductive quiescence and is beneficial in terms of cold hardiness, likely offering cold‐survival benefits during winter, it seems to come at a cost to immediate reproductive success (i.e., trade‐off). While cold‐developed and cold‐exposed males may survive the harsh winter period, their reduced sexual attractiveness and delayed reproductive ability could temporarily reduce their fitness, particularly in a competitive mating environment. However, with climate change, warmer winters and springs might allow cold‐acclimated winter males to reach fertility threshold and recover reproductive potential earlier.

In summary, the present study used a broad range of temperatures during both development and the adult stage to explore the reproductive potential of male *D. suzukii* at both low and high temperatures. This approach allows for a comprehensive analysis of thermal effects on reproductive traits with a TPC approach, providing a better understanding of these traits and helping to resolve potential inconsistencies in previous studies by considering a wide range of thermal conditions. It also provides crucial information on the reproductive potential of winter‐acclimated males (in reproductive quiescence) under both cold and optimal conditions. Cold acclimation allows survival but may impose a trade‐off, where reproductive function and competitiveness seem temporarily reduced. Indeed, the recovery of mating competitiveness was only partial and remained very mild. This understanding could help predict population dynamics in response to varying winter temperatures and broader climate change scenarios.

## Author Contributions

**Hervé Colinet:** conceptualization (equal), data curation (equal), formal analysis (equal), funding acquisition (equal), methodology (equal), project administration (equal), resources (equal), supervision (equal), visualization (equal), writing – original draft (equal), writing – review and editing (equal). **Coline Lehnhoff:** formal analysis (equal), investigation (equal), visualization (equal), writing – original draft (equal). **Bréa Raynaud‐Berton:** investigation (equal), supervision (equal), writing – review and editing (equal).

## Conflicts of Interest

The authors declare no conflicts of interest.

## Data Availability

All the data that support the findings of this study are available in the Dryad repository at the following permanent digital object identifier: https://doi.org/10.5061/dryad.tqjq2bw91.
